# Supplementary material for: High-Throughput Phenotyping for Agronomic Traits in Cassava Using Aerial Imaging
Source: Plants (Basel). 2024 Dec 25;14(1):32. doi: 10.3390/plants14010032 (PMC11723320; doi:10.3390/plants14010032)
Supplement: Supplementary file 1 [file plants-14-00032-s001.zip › plants-3225684-supplementary.pdf]

plants

Supplementary material

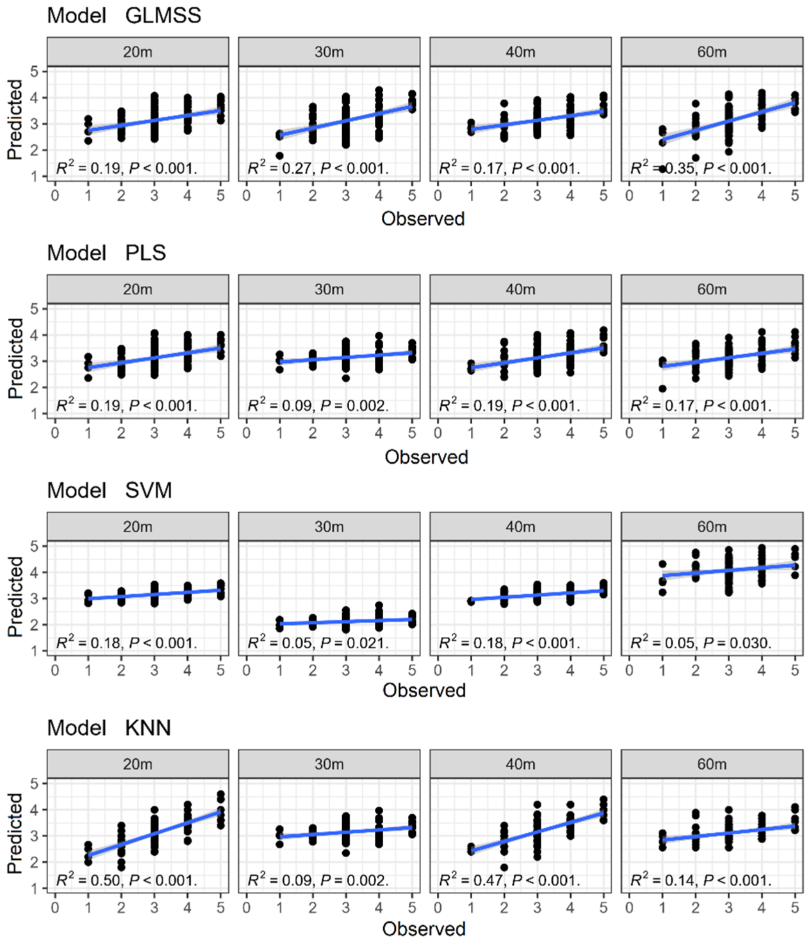

Figure S1. Relationship between observed and predicted values for cassava plant vigor. The predictions were made using four modeling approaches, incorporating cross-validation and selecting the best vegetation indices derived from RGB (Red, Green, Blue) and multispectral cameras obtained at four flight height (20 m, 30 m, 40 m, 60 m) from unmanned aerial vehicle. Generalized Linear Model with Stepwise Feature Selection (GLMSS), Partial Least Squares (PLS), Support Vector Machine (SVM), and K-Nearest Neighbor (KNN). R^2: Coefficient of Determination.

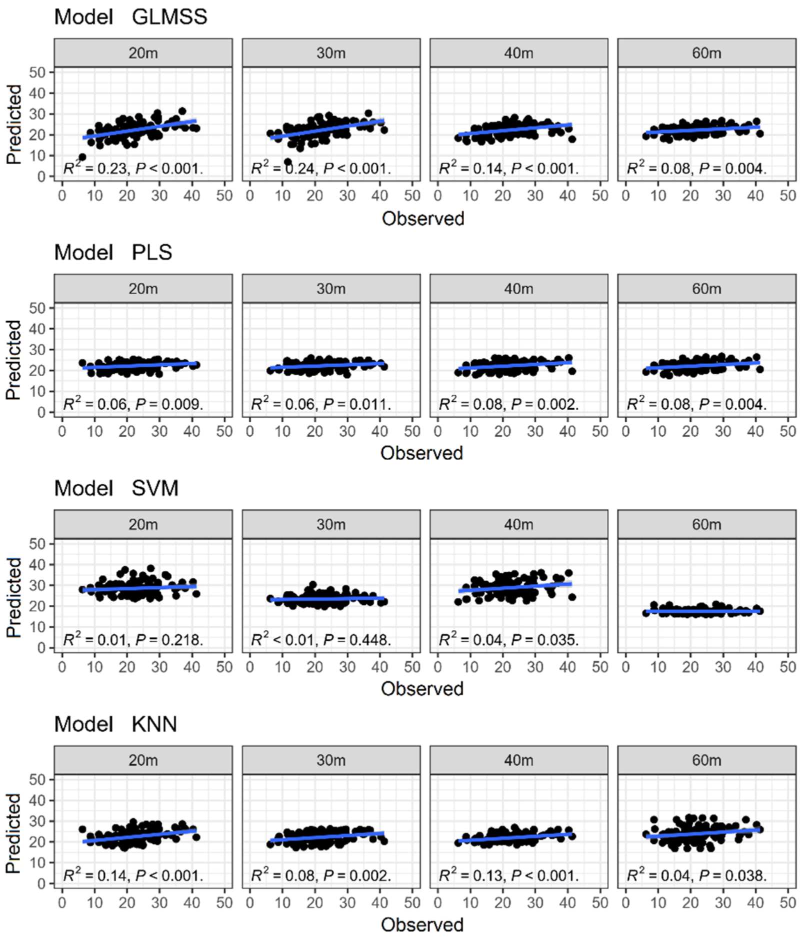

Figure S2. Relationship between observed and predicted values for cassava above-ground biomass yield. The predictions were made using four modeling approaches, incorporating cross-validation and selecting the best vegetation indices derived from RGB (Red, Green, Blue) and multispectral cameras obtained at four flight height (20 m, 30 m, 40 m, 60 m) from unmanned aerial vehicle. Generalized Linear Model with Stepwise Feature Selection (GLMSS), Partial Least Squares (PLS), Support Vector Machine (SVM), and K-Nearest Neighbor (KNN). R^2: Coefficient of Determination.

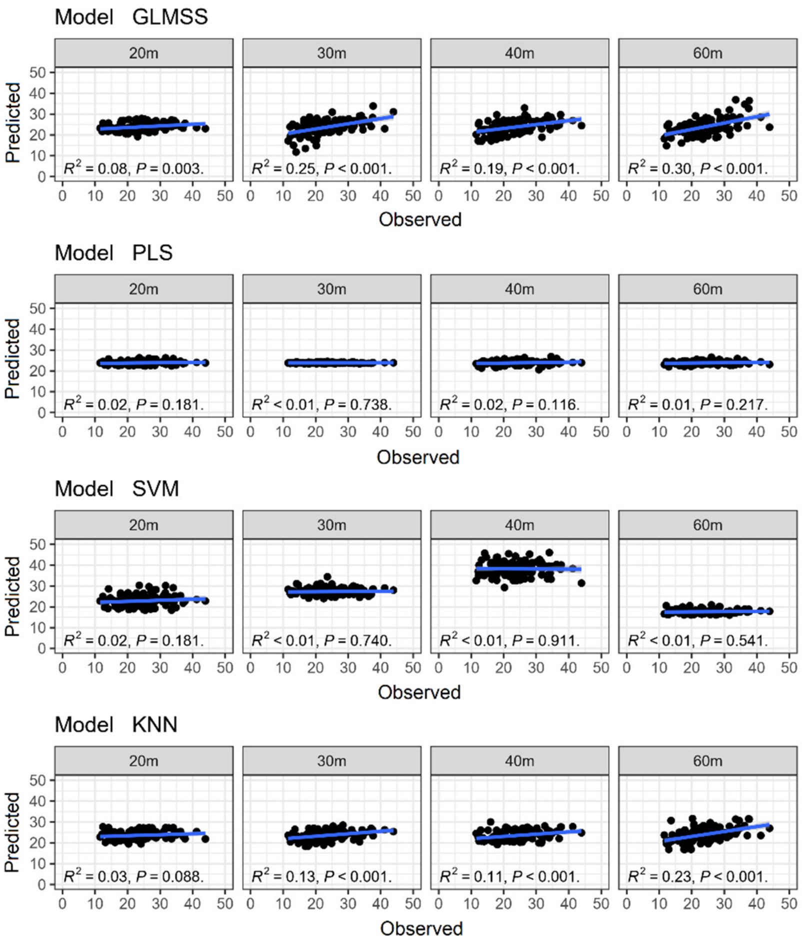

Figure S3. Relationship between observed and predicted values for cassava fresh root yield. The predictions were made using four modeling approaches, incorporating cross-validation and selecting the best vegetation indices derived from RGB (Red, Green, Blue) and multispectral cameras obtained at four flight height (20 m, 30 m, 40 m, 60 m) from unmanned aerial vehicle. Generalized Linear Model with Stepwise Feature Selection (GLMSS), Partial Least Squares (PLS), Support Vector Machine (SVM), and K-Nearest Neighbor (KNN). R^2: Coefficient of Determination.

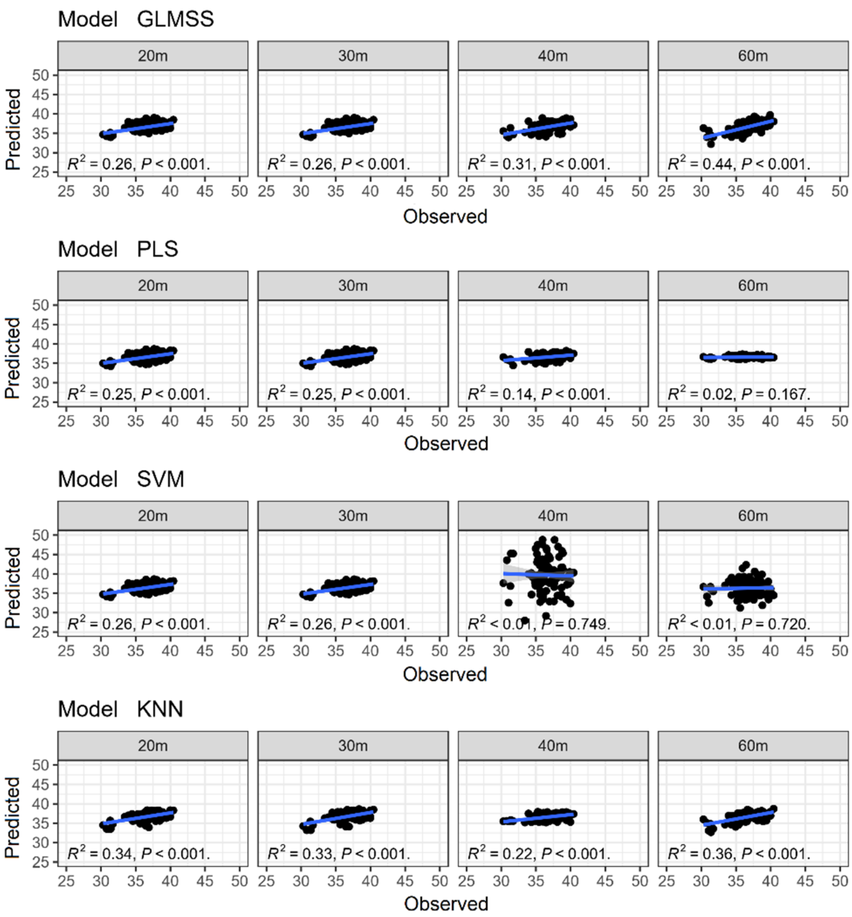

Figure S4. Relationship between observed and predicted values for the dry matter content in cassava roots. The predictions were made using four modeling approaches, incorporating cross-validation and selecting the best vegetation indices derived from RGB (Red, Green, Blue) and multispectral cameras obtained at four flight height (20 m, 30 m, 40 m, 60 m) from unmanned aerial vehicle. Generalized Linear Model with Stepwise Feature Selection (GLMSS), Partial Least Squares (PLS), Support Vector Machine (SVM), and K-Nearest Neighbor (KNN). R^2: Coefficient of Determination.

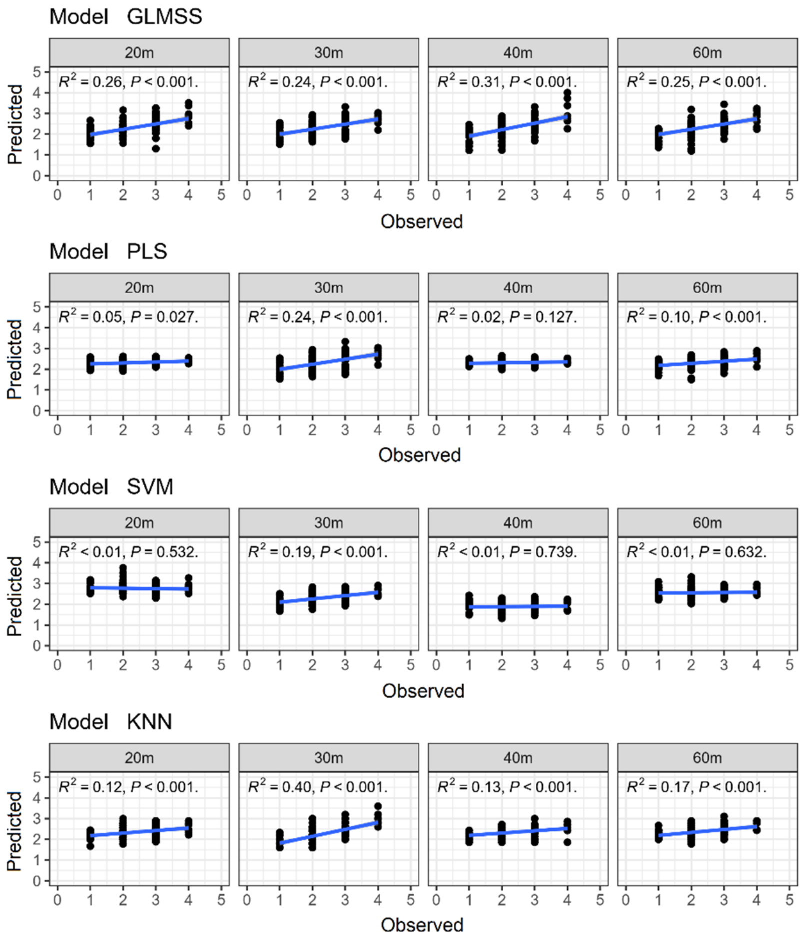

Figure S5. Relationship between observed and predicted values for leaf retention in cassava. The predictions were made using four modeling approaches, incorporating cross-validation and selecting the best vegetation indices derived from RGB (Red, Green, Blue) and multispectral cameras obtained at four flight height (20 m, 30 m, 40 m, 60 m) from unmanned aerial vehicle. Generalized Linear Model with Stepwise Feature Selection (GLMSS), Partial Least Squares (PLS), Support Vector Machine (SVM), and K-Nearest Neighbor (KNN). R^2: Coefficient of Determination.

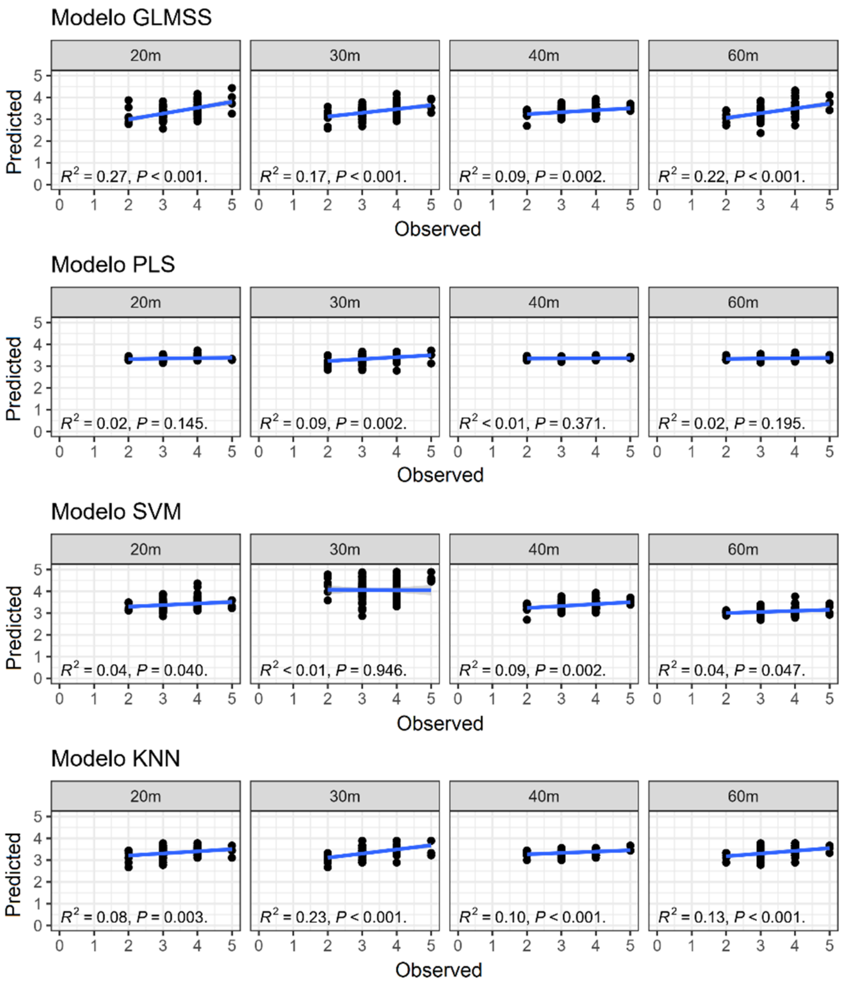

Figure S6. Relationship between observed and predicted values for leaf spots resistance in cassava. The predictions were made using four modeling approaches, incorporating cross-validation and selecting the best vegetation indices derived from RGB (Red, Green, Blue) and multispectral cameras obtained at four flight height (20 m, 30 m, 40 m, 60 m) from unmanned aerial vehicle. Generalized Linear Model with Stepwise Feature Selection (GLMSS), Partial Least Squares (PLS), Support Vector Machine (SVM), and K-Nearest Neighbor (KNN). R^2: Coefficient of Determination.

Table S1. Genealogy and classification of cassava genotypes based on cyanogenic compound (HCN) content.

Genotypes	HCN*	Type*	Male Parent	Female Parent
BGM-1683	6	Bitter	Unknown	Unknown
BGM-2095	6	Bitter	Unknown	Unknown
BGM-2097	4	Sweet	Unknown	Unknown
BGM-2151	6	Bitter	Unknown	Unknown
BR-18GS-003-45	5	Bitter	BGM-0046	BGM-0323
BR-18GS-011-28	3	Sweet	BGM-0128	BGM-0046
BR-18GS-031-11	4	Sweet	Unknown	BRS Tapioqueira
BR-18GS-031-6	3	Sweet	BGM-0661	BGM-2018
BR-18GS-054-40	3	Sweet	BGM-0888	BRS Tapioqueira
BR-18GS-054-48	6	Bitter	BGM-0888	BRS Tapioqueira
BR-18GS-056-12	4	Sweet	BGM-0991	BGM-0888
BR-18GS-056-9	3	Sweet	BGM-0991	BGM-0888
BR-18GS-076-13	6	Bitter	BGM-1253	BGM-2018
BR-18GS-080-24	6	Bitter	BGM-1268	BRS Tapioqueira
BR-18GS-084-17	4	Sweet	BGM-1332	BGM-2018
BR-18GS-084-2	4	Sweet	BGM-1332	BGM-2018
BR-18GS-084-22	4	Sweet	BGM-1332	BGM-2018
BR-18GS-084-34	4	Sweet	BGM-1332	BGM-2018
BR-18GS-111-21	4	Sweet	BGM-2044	BGM-0991
BR-18GS-111-23	4	Sweet	BGM-2044	BGM-0991
BR-18GS-111-45	3	Sweet	BGM-2044	BGM-0991
BR-18GS-111-54	4	Sweet	BGM-2044	BGM-0991
BR-18GS-111-57	3	Sweet	BGM-2044	BGM-0991
BR-18GS-111-6	5	Bitter	BGM-2044	BGM-0991
BR-18GS-111-78	4	Sweet	BGM-2044	BGM-0991
BR-18GS-113-4	4	Sweet	BGM-2044	BGM-2127
BR-18GS-128-24	5	Bitter	Unknown	BGM-1760
BR-18GS-131-11	4	Sweet	Unknown	BRS Tapioqueira
BR-18GS-134-29	2	Sweet	BRS Tapioqueira	BGM-0818
BRS-Dourada	4	Sweet	Unknown	Unknown
BRS-Gema-de ovo	3	Sweet	Unknown	Unknown
BRS-Jari	3	Sweet	BRS Dourada	BGM-1721
BRS-Kiriris	4	Sweet	Unknown	BGM-0921
BRS-Novo Horizonte	6	Bitter	BGM-1727	BGM-0116
BRS-Poti-Branca	6	Bitter	Unknown	SM-807

*HCN was determined using the picrate test, where three to four root samples from each genotype were placed in a test tube containing toluene solution, alkaline picrate (color reagent), and filter paper. After 24 hours, the color of the filter paper was evaluated using a color scale ranging from 1 to 9. The average score was used to classify cassava genotypes as sweet (scores 1 to 4), intermediate (score 5), or bitter (scores > 6).

Table S2. Spectral resolution of RGB and Micasense RedEdge-M bands for remote sensing applications.

Band Number	Band type	Wavelength (nm)	Band Width (nm)
1	Blue	475	20
2	Green	560	20
3	Red	668	10
4	Red Edge	717	10
5	NIR (Near Infrared)	840	40
